# Controlling self-assembly of diphenylalanine peptides at high pH using heterocyclic capping groups

**DOI:** 10.1038/srep43947

**Published:** 2017-03-08

**Authors:** Adam D. Martin, Jonathan P. Wojciechowski, Andrew B. Robinson, Celine Heu, Christopher J. Garvey, Julian Ratcliffe, Lynne J. Waddington, James Gardiner, Pall Thordarson

**Affiliations:** 1 School of Chemistry, The Australian Centre for Nanomedicine and the ARC Centre for Convergent Bio-Nano Science and Technology, University of New South Wales, Sydney, NSW, 2052, Australia; 2 Biomedical Imaging Facility (BMIF), Mark Wainwright Analytical Centre, University of New South Wales, Sydney NSW, 2052, Australia; 3 Australian Nuclear Science and Technology Organisation, New Illawarra Rd, Lucas Heights, NSW, 2231, Australia; 4 CSIRO Manufacturing, Bayview Ave, Clayton, Victoria 3168, Australia

## Abstract

Using small angle neutron scattering (SANS), it is shown that the existence of pre-assembled structures at high pH for a capped diphenylalanine hydrogel is controlled by the selection of N-terminal heterocyclic capping group, namely indole or carbazole. At high pH, changing from a somewhat hydrophilic indole capping group to a more hydrophobic carbazole capping group results in a shift from a high proportion of monomers to self-assembled fibers or wormlike micelles. The presence of these different self-assembled structures at high pH is confirmed through NMR and circular dichroism spectroscopy, scanning probe microscopy and cryogenic transmission electron microscopy.

Hydrogels composed of short peptides have rapidly gained attention over the past ten years, owing to their high biocompatibility, ease of synthesis and tunability^1^,^2^,^3^. Due to their resemblance to the native extracellular matrix, these materials have been used to culture various cell lines, release drug molecules in a controlled manner and promote neural outgrowth^4^,^5^,^6^,^7^,^8^. While the amino acid sequence used plays a major part in determining the physical and chemical properties of the hydrogel, short peptides often require an aromatic capping group at their N-terminus to induce gelation^9^,^10^. The hydrophobic interactions associated with this capping group combine with hydrogen bonding of the peptide backbone to drive self-assembly in these systems. A variety of functional capping groups have been employed, from photo-switchable moieties, to fluorophores, to heterocycles^11^,^12^,^13^,^14^. These capping groups play as important a role in the final hydrogel structure as the amino acid sequence selected.

Small angle neutron scattering (SANS) is an extremely useful tool when studying the native environment of a hydrogel. Whereas microscopy techniques such as transmission electron microscopy (TEM) and atomic force microscopy (AFM) are liable to suffer from the presence of sample preparation artefacts, small angle scattering is conducted on the native hydrogel, providing an accurate statistical three dimensional perspective of the actual hydrogel structure. SANS measurements have been extensively used to probe the native structure of peptide-based hydrogels, most often on hydrogel samples which already exhibit a well-defined network^15^,^16^,^17^,^18^,^19^,^20^,^21^,^22^,^23^,^24^,^25^,^26^. Less well studied is small angle scattering on transitions within gelators; however there are examples of SANS being used to examine the transition of a hexapeptide from ribbons to fibers^27^ and using SANS to look at the evolution of a pyromellitamide organogel over several days^28^. Curiously, there are limited examples of small angle scattering being used to probe the early stages of gelation in a supramolecular peptide hydrogel^15^,^29^, perhaps owing to difficulties in obtaining usable time resolution.

Herein, we probe the early stages of gelation using small angle neutron scattering and show that the choice of N-terminal heterocyclic capping group plays a vital role in determining the mechanism of self-assembly for the dipeptide hydrogels indole-diphenylalanine **1** and carbazole-diphenylalanine **2**, which is likely controlled through the presence of pre-formed aggregates at high pH. From analysis of the SANS scattering profile of **2**, it can be seen that minimal changes occur over 4 h, suggesting that fibers or wormlike micelles spontaneously form at high pH. This is supported through cryogenic TEM (*cryo*-TEM) and AFM imaging. This extends the applicability of findings previously established for naphthalene-capped dipeptide hydrogels^30^, by showing that the self-assembly of N-capped short peptides at high pH is more than likely controlled by the hydrophobicity of the heterocyclic capping group^31^.

## Results and Discussion

**Figure 1:**
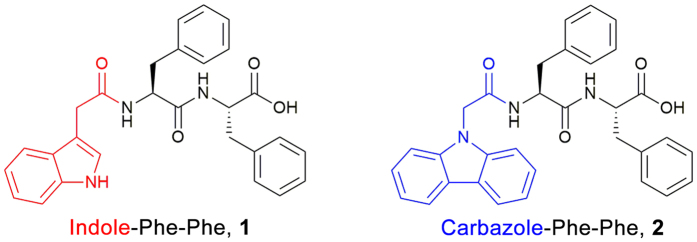
Gelators used in this study, containing the same peptide sequence but differing in their N-terminal capping group.

Indole-diphenylalanine **1** and carbazole-diphenylalanine **2** (Fig. 1) were synthesized using solid phase peptide synthesis, employing a 2-chlorotritylchloride resin and standard Fmoc chemistry (see Supplementary Information for details on synthesis, preparation of gels, SANS, rheology, spectroscopy and microscopy measurements). Yields and characterization of these dipeptides and their subsequent hydrogels were in agreement with that previously reported^32^,^33^. These gelators were selected owing to their vastly different hydrogel stiffnesses (3 × 10^5^ Pa for **1** vs. 600 Pa for **2**)^32^,^33^, which arises from a very different fiber arrangement within the hydrogel network. Indole-diphenylalanine **1** is composed of thick, bundled fibers (>100 nm) with minimal cross-linking^32^, whereas carbazole-diphenylalanine **2** consists of highly interwoven 3 nm fibers^33^. We theorized that this difference in gel network was likely due to different self-assembly processes occurring for each gelator, something which we then examined in greater detail.

**Figure 2:**
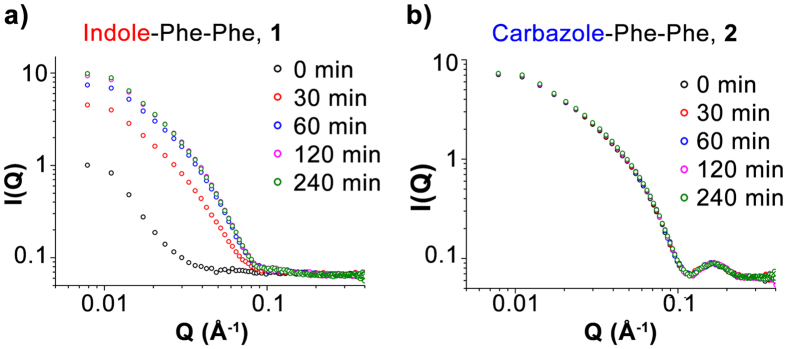
Evolution of SANS scattering patterns for (**a**) indole-diphenylalanine **1** and (**b**) carbazole-diphenylalanine **2** over a four hour period. *Time = 0 min represents the sol state at pD = 9*, before any addition of GdL, and hydrogels were prepared in D_2_O at a concentration of 1% (w/v).

To trigger gelation in these dipeptides, a pH switching mechanism using glucono-δ-lactone (GdL) was employed^34^. As GdL dissolves rapidly yet hydrolyses to gluconic acid slowly, this allows for a slow, controlled and homogenous pH drop throughout the sample, compared to the use of mineral acids. For time resolved neutron scattering measurements (Fig. 2), hydrogels were prepared in D_2_O (at pD 9) and quickly transferred to a demountable titanium SANS cell which was then placed in a 20 position sample changer and measurements undertaken. For pre-formed gels, the samples were allowed to age in the demountable cell overnight to reach a fully formed network structure before measurements were performed. All hydrogels were formed at 1% (w/v). SANS measurements were performed over a *q* range of 0.01–0.4 Ǻ^−1^, which corresponds to probing a length scale of 1.6–63 nm. It should be noted that for these measurements, time zero (i.e. t = 0 min, pD = 9) corresponds to the peptide sol as confirmed by rheological measurements (see Supplementary Fig. S1), before the addition of any GdL to the gelator.

For indole-diphenylalanine **1** (Fig. 2a), a distinct difference in scattering profile between the sol (t = 0 min, pD = 9) and gel (t = 240 min, pD = 4) is evident. The evolution of this scattering profile over four hours is shown in Fig. 1a, with the majority of the changes occurring in the first hour. Using SasView^35^, the data was fitted to a cylindrical model, which from existing AFM and TEM measurements on this network^32^, is physically realistic due to the limited cross-linking between fibers. It can be seen in Table 1 that over the first hour, whilst the radius of the fibers decrease slightly, possibly due to a dynamic rearrangement process, the length of the fibers dramatically decreases. This is to be expected, as the growth of an interwoven gel network will result in a greater frequency of intersecting fibers, thus resulting in a lower effective fiber length.

For carbazole-diphenylalanine, **2** (Fig. 2b), there is no such evolution of the scattering pattern over time. There is a slight change between sol and pre-formed gel scattering patterns at low *q*, suggesting a change in the large-scale network, which is consistent with network formation on a larger scale than what could be probed in these measurements. However, when fit to a flexible cylinder model, the majority of the scattering pattern shows no difference, and this is also reflected in the outputs obtained from this sample (Table 1 and see Supplementary Figs S2 and S3). The presence of a peak in the sol and gel scattering patterns at *q* = 0.19 Ǻ^−1^ suggests a high fiber monodispersity. The lack of change in the scattering profile, from the sol to gel formation over 4 h means that self-assembled structures similar to those comprising the hydrogel network must be present at high pH.

Attempts were made to visualize the evolution of hydrogel networks of **1** and **2** using transmission electron microscopy. Both *cryo*-TEM and normal TEM using negative staining were performed. Snapshots of hydrogel formation for **1** and **2** were taken each hour over four hours, in order to correlate the timescale of network evolution with that observed through SANS.

**Figure 3:**
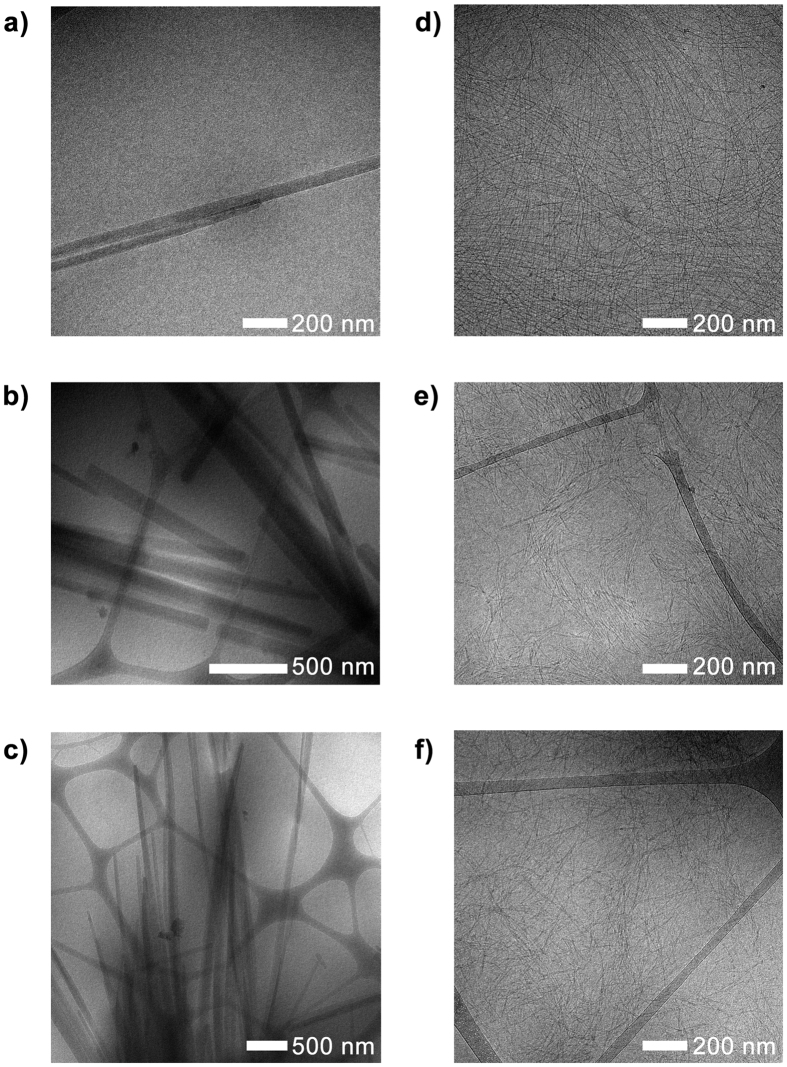
c*ryo*-TEM snapshots of gelation for indole-diphenylalanine **1**, taken at (**a**) 1 h, (**b**) 2 h, and (**c**) 4 h; and for carbazole-diphenylalanine **2**, taken at (**d**) 1 h, (**e**) 2 h, and (**f**) 4 h. Scale bars are as shown for each image.

For hydrogels of indole-diphenylalanine **1** after one hour, only long, sparse fibers are visible (Fig. 3a). Over the course of four hours, these fibers laterally aggregate, forming bundles (Fig. 3b and c). The changes seen in these *cryo*-TEM measurements cannot be directly correlated to the results obtained from SANS, as these >100 nm fibers fall outside the *q* range measured. However, taken together, the results indicate a hierarchical self-assembly process occurring across multiple length scales.

For carbazole-diphenylalanine **2**, even after only one hour, a fibrous network consisting of long, intersecting fibers is visible (Fig. 3d). This network does not change notably over time (Fig. 3e and f, however this can be better visualized using the negative stain TEM images, see Supplementary Fig. S4), with the fiber radius from *cryo*-TEM corresponding very well to that determined form SANS.

The *cryo*-TEM data for both gelators, taken together with SANS results, suggests a different mechanism of self-assembly for each gelator. As the amino acid sequence of each gelator is identical, this must be controlled by the choice of capping group. That the more hydrophilic indole capping group does not form self-assembled fibers or wormlike micelles at high pH shows that previous design rules established for naphthalene-capped short peptides^31^ appear applicable to heterocycle-capped peptides, with the more hydrophobic carbazole capping group yielding self-assembled structures at high pH.

Previously, the formation of wormlike micelles in the self-assembly of naphthalene-capped diphenylalanine peptides at high pH has been reported^30^. Addition of a variety of salts triggered gelation in these wormlike micelle-based systems^15^. The effect of electron donating and withdrawing substituents at the 6-position of the naphthyl group was investigated^31^, with hydrophobicity shown to be the main driving force behind self-assembly at high pH. The SANS and *cryo*-TEM data presented in our work seem to support this model in terms of the role of hydrophobicity in controlling self-assembly at high pH.

**Figure 4:**
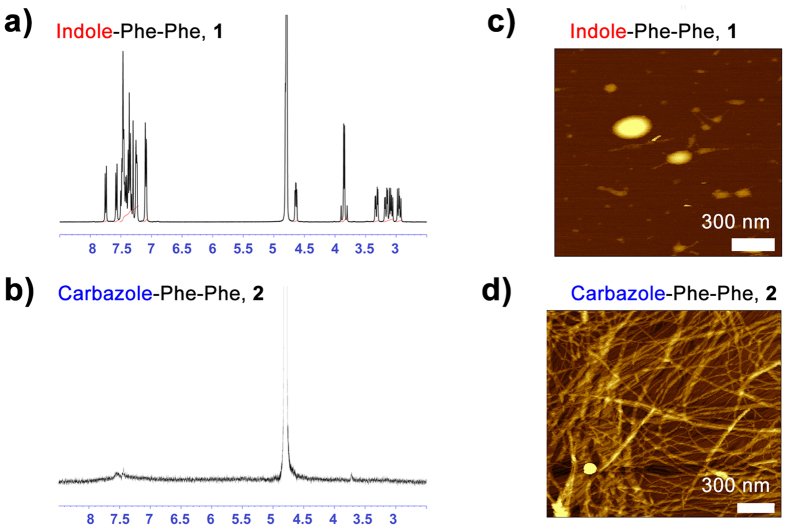
^1^H NMR (400 MHz, D_2_O + NaOD, pD = 10) of (**a**) indole-diphenylalanine **1** sol and (**b**) carbazole-diphenylalanine **2** sol (**b**) with the corresponding AFM of (**c**) indole-diphenylalanine **1** sol at 0.1% (w/v) and (**d**) carbazole-diphenylalanine 2 sol at 0.01% (w/v). Scale bars represent 300 nm.

Now that the role of heterocyclic capping group on self-assembly at high pH has been established, we sought to define the nature of these high pH self-assembled structures. ^1^H NMR of the peptides dissolved in basic D_2_O (pD 10) was performed, and shows a sharp set of signals for indole-diphenylalanine, **1** (Fig. 4a), which matches its spectrum in DMSO^33^. This suggests that in basic environments, **1** is well solvated and a high proportion of monomers exist. This may also indicate the presence of self-assembled structures undergoing fast exchange processes. Carbazole-diphenylalanine **2** in basic D_2_O, however, shows no sharp NMR signals aside from the solvent peak (Fig. 4b) with all the remaining proton resonances broadened out over the entire baseline. Lowering the pD to 9, yielded very similar spectra (see Supplementary Fig. S5). This is consistent with an assembly of the molecule into a “frozen-in” supramolecular structure exhibiting limited exchange and consequent effect of immobility on spin-spin relaxation.

Atomic force microscopy (AFM) was performed on the peptide sols, spread coated onto a mica substrate. For **1**, at all concentrations that were imaged (0.1 to 1% (w/v)), only amorphous aggregates were observed (Fig. 4c). However for **2**, it can clearly be seen that fibers are present at concentrations as low as 0.01% (w/v) (Fig. 4d). Both large and small fibers can be seen, with the small fibers having a diameter of 3–4 nm, which is consistent with AFM previously performed on hydrogels of **2**^33^. The presence of larger fibers is most likely due to aggregation during sample drying. These images clearly confirm that self-assembled structures, either fibers or wormlike micelles, are present in sols of **2** but not **1**.

Shear rate measurements have previously been used in conjunction with imaging to confirm the presence of wormlike micelles for short peptides^30^. Here, shear rate sweeps were performed on 1% (w/v) solutions of **1** and **2**, which both show shear thinning behaviour at high shear rates and possess viscosities at 1 s^−1^ of 2 and 10 mPa∙s for **1** and **2**, respectively. This implies that **2** may exist at high pH as wormlike micelles. This is less likely for **1**, therefore dynamic light scattering (DLS) was conducted on a 1% (w/v) sol of **1** to check for the presence of aggregates. Using DLS, a peak centred at approximately 150 nm was observed, confirming the presence of aggregates. It should be noted however, that the standard deviation of this peak was significant (66 nm), which is either suggestive of polydispersity of the aggregates or dynamic behaviour of the aggregates (i.e. rapid exchange of monomers between the aggregates and the solution phase). Based upon the aforementioned AFM and NMR measurements, it is likely that both of these processes are present for sols of **1** at high pH.

## Conclusions

In conclusion, we show that the selection of N-terminal capping group on a short peptide plays a crucial role in the self-assembly mechanism for diphenylalanine–based hydrogels. Indole-diphenylalanine **1** forms dynamic small aggregates in basic water, before self-assembling upon the addition of a pH trigger to give a highly bundled fiber network. Replacing indole with the more hydrophobic carbazole resulted in the formation of fibers or wormlike micelles at high pH. A suite of complementary techniques such as SANS, *cryo*-TEM, AFM, rheology and ^1^H NMR have been used to elucidate these different self-assembly pathways across different timescales. These results are in line with earlier studies on naphthyl-based dipeptides^30^, where self-assembly at high pH appears to be controlled by the hydrophobicity of the capping group for these peptides. This has important implications for the future rational design of responsive short peptide hydrogels bearing different heterocyclic capping groups. Controlling the presence of pre-formed aggregates is especially useful, as in these systems gelation can be triggered by mixing with buffers or cell media, with salts in the media screening charges on these aggregates. This will result in hydrogels in which formation does not have to be triggered by an acid, which is advantageous for potential applications in cell culture and biomedicine.

## Additional Information

**How to cite this article:** Martin, A. D. *et al*. Controlling self-assembly of diphenylalanine peptides at high pH using heterocyclic capping groups. *Sci. Rep.* **7**, 43947; doi: 10.1038/srep43947 (2017).

**Publisher's note:** Springer Nature remains neutral with regard to jurisdictional claims in published maps and institutional affiliations.

## Supplementary Material

Supplementary Information

## Figures and Tables

**Table 1:** Summary of outputs from fitting the SANS data obtained for **1** and **2** to a flexible cylinder model.

Time (min)	Indole-diphenylalanine, 1		Carbazole-diphenylalanine, 2	
	Radius (Ǻ)	Length (Ǻ)	Radius (Ǻ)	Length (Ǻ)
0	90.5 ± 1.9	(5.3 ± 0.9) x 10^4^	33.34 ± 0.1	382 ± 19
30	44.5 ± 0.2	4493 ± 574	33.34 ± 0.1	422 ± 21
60	42.4 ± 0.1	2093 ± 399	33.34 ± 0.1	452 ± 24
120	42.5 ± 0.1	2091 ± 401	33.34 ± 0.1	457 ± 24
240	42.4 ± 0.1	2092 ± 394	33.34 ± 0.1	458 ± 25

Large uncertainties appear for indole-diphenylalanine **1** fiber lengths due to the large distribution of fiber sizes.

## References

[b1] Adler-Abramovich L. & Gazit E. The physical properties of supramolecular peptide assemblies: from building block association to technological applications. Chem. Soc. Rev. 43, 6881–6893 (2014). 25099656 10.1039/c4cs00164h

[b2] Weiss R. G. The past, present, and future of molecular gels: What is the status of the field, and where is it going? J. Am. Chem. Soc. 136, 7519–7530 (2014). 24836858 10.1021/ja503363v

[b3] De Santis E. & Ryadnov M. G. Peptide self-assembly for nanomaterials: the old new kid on the block. Chem. Soc. Rev. 44, 8288–8300 (2015). 26272066 10.1039/c5cs00470e

[b4] Tao K., Levin A., Adler-Abramovich L. & Gazit E. Fmoc-modified amino acids and short peptides: simple, bio-inspired building blocks for the fabrication of functional materials. Chem. Soc. Rev. 45, 3935–3953 (2016). 27115033 10.1039/c5cs00889a

[b5] Skilling K. J. . Insights into low molecular mass organic gelators: a focus on drug delivery and tissue engineering applications. Soft Matter 10, 237–256 (2014). 24651822 10.1039/c3sm52244j

[b6] Newcomb C. J. . Cell death versus cell survival instructed by supramolecular cohesion of nanostructures. Nat. Commun. 5, 3321 (2014). 24531236 10.1038/ncomms4321 PMC3982852

[b7] Martin C. . Injectable peptide hydrogels for controlled release of opiods. Med. Chem. Comm. 7, 542–549 (2016).

[b8] Bibian M. . Rational design of a hexapeptide hydrogelators for controlled release drug delivery. J. Mater. Chem. B 3, 759–765 (2015). 10.1039/c4tb01294a 32262166

[b9] Fichman G. & Gazit E. Self-assembly of short peptides to form hydrogels: Design of building blocks, physical properties and technological applications. Acta Biomater. 10, 1671–1682 (2014). 23958781 10.1016/j.actbio.2013.08.013

[b10] Hsu S.-M., Lin Y.-C., Chang J.-W., Liu Y.-H. & Lin H.-C. Intermolecular interactions of a phenyl/perfluorophenyl pair in the formation of supramolecular nanofibers and hydrogels. Angew. Chem. Int. Ed. 53, 1921–1927 (2014). 10.1002/anie.201307500 24420005

[b11] Draper E. R., Eden E. G. B., McDonald T. O. & Adams D. J. Spatially resolved multicomponent gels. Nat. Chem. 7, 848–852 (2015). 26391086 10.1038/nchem.2347

[b12] Wang H. . Biocompatible supramolecular nanofibrous hydrogel for long-term cell tracking and tumor imaging applications. Sci. Rep. 5, 16680 (2015). 26573372 10.1038/srep16680 PMC4647837

[b13] Martin A. D., Wojciechowski J. P., Warren H., in het Panhuis M. & Thordarson P. Effect of heterocyclic capping groups on the self-assembly of a dipeptide hydrogel. Soft Matter 12, 2700–2707 (2016). 26860207 10.1039/c6sm00025h

[b14] Martin A. D., Wojciechowski J. P., Bhadbhade M. M. & Thordarson P. A capped dipeptide that simultaneously exhibits gelation and crystallization behavior. Langmuir 32, 2245–2250 (2016). 26890360 10.1021/acs.langmuir.5b03963

[b15] Cardoso A. Z. . Linking micellar structures to hydrogelation for salt-triggered dipeptide hydrogelators. Soft Matter 12, 3612–3621 (2016). 26963370 10.1039/c5sm03072b

[b16] Morris K. L. . Chemically programmed self-sorting of gelator networks. Nat. Commun. 4, 1480 (2013). 23403581 10.1038/ncomms2499

[b17] Maslovskis A. . Self-assembling peptide/thermoresponsive polymer composite hydrogels: effect of peptide-polymer interactions on hydrogel properties. Langmuir 30, 10471–10480 (2014). 25095719 10.1021/la502358b

[b18] Zhao Y. . Tuning the self-assembly of short peptides via sequence varations. Langmuir 29, 13457–13464 (2013). 24090051 10.1021/la402441w

[b19] Rexeisen E. L. . Self-assembly of fibronectin-mimetic peptide-amphiphile nanofibers. Langmuir 26, 1953–1959 (2010). 19877715 10.1021/la902571q

[b20] Guilbaud J.-B. & Saiani A. Using small angle scattering (SAS) to structurally characterize peptide and protein self-assembled materials. Chem. Soc. Rev. 40, 1200–1211 (2011). 21113529 10.1039/c0cs00105h

[b21] Baral A. . A peptide-based mechano-sensitive, proteolytically stable hydrogel with remarkable antibacterial properties. Langmuir 32, 1836–1845 (2016). 26818698 10.1021/acs.langmuir.5b03789

[b22] Baral A. . Time dependent gel to gel transformation of a peptide-based supramolecular gelator. Soft Matter 11, 4944–4951 (2015). 26016677 10.1039/c5sm00808e

[b23] De Leon-Rodriguez L. M. . A peptide hydrogel derived from a fragment of human cardiac troponin-C. Chem. Commun. 52, 4056–4059 (2016). 10.1039/c6cc00209a 26892840

[b24] Nagarkar R. P., Hule R. A., Pochan D. J. & Schneider J. P. De novo design of strand-swapped β-hairpin hydrogels. J. Am. Chem. Soc. 130, 4466–4474 (2008). 18335936 10.1021/ja710295t

[b25] Hule R. A., Nagarkar R. P., Hammouda B., Schneider J. P. & Pochan D. J. Dependence of self-assembled peptide hydrogel network structure on local fibril nanostructure. Macromolecules 42, 7137–7145 (2009). 21566682 10.1021/ma9003242 PMC3091019

[b26] Branco M. C., Nettesheim F., Pochan D. J., Schneider J. P. & Wagner N. J. Fast dynamics of semiflexible chain networks of self-assembled peptides. Biomacromolecules 10, 1374–1380 (2009). 19391585 10.1021/bm801396e PMC2738993

[b27] Zhao Y., Deng L., Wang J., Xu H. & Lu J. R. Solvent controlled structural transition of KI_4_K self-assemblies: from nanotubes to nanofibrils. Langmuir 31, 12975–12983 (2015). 26540520 10.1021/acs.langmuir.5b02303

[b28] Jamieson S. A. . Small angle neutron scattering (SANS) studies on the structural evolution of pyromellitamide self-assembled gels. Langmuir 30, 13987–13993 (2014). 25361640 10.1021/la502546n

[b29] Cardoso A. Z. . The influence of the kinetics of self-assembly on the properties of dipeptide hydrogels. Faraday Discuss. 166, 101–116 (2013). 24611271 10.1039/c3fd00104k

[b30] Chen L. . Salt induced hydrogelation of functionalized-dipeptides at high pH. Chem. Commun. 47, 12071–12073 (2011). 10.1039/c1cc15474e 22005767

[b31] Chen L., McDonald T. O. & Adams D. J. Salt-induced hydrogels from functionalized dipeptides. RSC Adv. 3, 8714–8720 (2013).

[b32] Martin A. D., Robinson A. B., Mason A. F. & Thordarson P. Exceptionally strong hydrogels through self-assembly of an indole-capped dipeptide. Chem. Commun. 50, 15541–15544 (2014). 10.1039/c4cc07941h 25354784

[b33] Martin A. D., Robinson A. B. & Thordarson P. Biocompatible small peptide super-hydrogelators bearing carbazole functionalities J. Mater. Chem. B. 3, 2277–2280 (2015). 10.1039/c5tb00067j 32262056

[b34] Adams D. J. . A new method for maintaining homogeneity during liquid-hydrogel transitions using low molecular weight hydrogelators. Soft Matter 5, 1856–1862 (2009).

[b35] *SasView for Small Angle Scattering Analysis*, http://www.sasview.org/, accessed 01/05/2016.

