# Supplementary material for: Controlling self-assembly of diphenylalanine peptides at high pH using heterocyclic capping groups
Source: Sci Rep. 2017 Mar 8;7:43947. doi: 10.1038/srep43947 (PMC5341053; doi:10.1038/srep43947)
Supplement: Supplementary Information [file srep43947-s1.pdf]

Supplementary information

Controlling self-assembly of diphenylalanine peptides at high pH using heterocyclic capping groups

Adam D. Martin,*[a] Jonathan P. Wojciechowksi,[a] Andrew B. Robinson,[a] Celine Heu,[a,b] Christopher J. Garvey,[c] Julian Ratcliffe,[d] Lynne J. Waddington,[d] James Gardiner,[d] Pall Thordarson*[a]

[a] School of Chemistry, The Australian Centre for Nanomedicine and the ARC Centre for Convergent Bio-Nano Science and Technology, University of New South Wales, Sydney, NSW, Australia, 2052

[b] Biomedical Imaging Facility, Mark Wainwright Analytical Centre, University of New South Wales, Sydney, NSW, Australia, 2052

[c] Australian Nuclear Science and Technology Organisation, New Illawarra Rd, Lucas Heights, NSW, Australia, 2231.

[d] CSIRO Manufacturing, Bayview Ave, Clayton, Victoria 3168, Australia.

E-mails: adam.martin2@unsw.edu.au and p.thordarson@unsw.edu.au; Fax: +61-2-9385-6141; Tel: +61-2-9385-4478.

Contents:

Synthesis of compounds **1** and **2** S2

Preparation of hydrogels S2

SANS measurements S2

cryo-TEM measurements S2

Circular dichroism measurements S3

Rheology measurements S3

AFM measurements S3

NMR measurements S3

Figure S1 – Frequency sweep of sols of **1** and **2** S4

Figure S2 – Fits of SANS data of **1** to cylindrical model S4

Figure S3 – Fits of SANS data of **2** to flexible cylinder model S5

Figure S4 – Negative stain TEM of **2** after 1h and 4h S5

Figure S5 – ^{1}H NMR of **2** at pD = 9 S6

References S6

Synthesis of compounds 1 and 2

Using reported methods, indole-diphenylalanine **1**[1] and carbazole-diphenylalanine **2**[2] were synthesised using solid phase peptide synthesis, with the characterisation data in good agreement with those reports.

Preparation of hydrogels

1.5 equivalents of 0.1 M sodium hydroxide was added to the peptide of interest and milliQ water added to make the suspension up to the required concentration (either 0.5 or 1% (w/v)). This suspension was briefly sonicated (30-60 seconds total, in intervals of 5 seconds) at low power using an ultrasonic bath until homogenous, upon which time 3 molar equivalents of glucono-δ-lactone was added to lower the pH, resulting in gelation.

SANS measurements

Hydrogels were prepared according to the above method and transferred as quickly as possible to a demountable titanium cell of 1 mm path length. Here, the peptides were dissolved as above, GdL added and the solution transferred to the demountable cell, where gelation occurs over time. Typical delays from the addition of GdL to data collection were approximately four minutes. Measurements were performed at the QUOKKA beamline at ANSTO, at 25 °C, at a detector distance of 2 m, which allowed for a time resolution of approximately 10 mins per measurement and a q range of 0.008 – 0.41 Å to be covered. Neutrons of wavelength 5 Å with a wavelength spread characterised by a full width half maximum of 12%. The scattered data was corrected for the background, empty cell scattering and the sensitivity of the individual detector pixels. The data was reduced using IgorPro software[3] employing NIST macros specific to QUOKKA[4] to an absolute intensity scale and modelled using SasView.[5]

cryo-TEM measurements

All samples were prepared on 200-mesh copper grids coated with perforated carbon film (Lacey carbon film: ProSci-Tech, Qld, Australia). The grids were glow discharged under an inert nitrogen atmosphere to render them hydrophilic. Hydrogels were prepared as described above at 1% (w/v). After allowing the gels to set for 1 h, 2 h, 3 h, or 4 h, the gel sample (10 µL),diluted (1:4) and vortexed. This solution (5 µL) was then added to a grid secured in the vitrification apparatus and allowed to adsorb for approximately 20 seconds and excess liquid removed through blotting. A specialised, in-house vitrification apparatus was used to prepare the samples. Images were obtained using a Tecnai 12 Transmission Electron Microscope and Gatan 626 cryoholder (Gatan, Pleasanton, CA, USA) with an FEI Eagle 4k x 4k CCD camera (FEI, Eindhoven, Netherlands) and AnalySIS camera control software. The operating voltage used was 120 kV and low dose procedures were followed using an electron dose of 10 electrons/Å^2.

TEM measurements

Grids and samples were prepared as above for *cryo*-TEM measurements. To each grid diluted gel solution (5 µL) was added by pipette. After being left to adsorb for approximately 20

seconds the liquid was removed by gently exposing it to the corner of a piece of Whatman 541 filter paper. A solution of 2% phosphotungstic acid (5 µL, pH 7.2) was added by pipette and left to adsorb for approximately 20 seconds before being similarly removed. Images were obtained using a Tecnai 12 Transmission Electron Microscope with a CCD camera and AnalySIS camera control software. The operating voltage was 120 kV and low dose procedures were followed using and electron dose of 10 electrons/Å^2.

Circular dichroism measurements

CD measurements were performed using a ChirascanPlus CD spectrometer, with data collected between wavelengths of 180 – 500 nm with a bandwidth of 1 nm, sample ratio of 0.1 s/point and step of 1 nm. In a typical experiment, 1% (w/v) peptide sols were prepared as above and diluted 1:20 (v/v) in water. Temperature was kept constant at 20 °C and all experiments were repeated three times and averaged into a single plot.

Rheology measurements

Rheological measurements were performed on an Anton Paar MCR 302 rheometer using a 25 mm stainless steel parallel plate geometry configuration and analysed using RheoPlus v3.61 software. Typical rheology measurements involved casting 550 µL of a 1% (w/v) sol onto one of the stainless steel plates, lowering the other plate to the measurement position, and allowing two hours for the gel to form *via* the pH switch method described above. A Peltier temperature control hood and solvent trap was used to reduce evaporation and maintain a temperature of 25 °C for frequency and amplitude sweeps. Frequency sweeps were performed with a log ramp frequency (f) = 0.01 – 10 Hz and constant strain (γ) = 0.5%. The rheology plots displayed are an average of at least three repeats for each point and error bars denote two standard deviations from the log-averaged mean.

AFM measurements

Peptide sols of **1** and **2** were prepared using the pH switch method described above and one drop of the hydrogel solutions was cast onto a freshly cleaved mica substrate, followed by spreading of the drop over the mica using a glass slide, with the excess liquid wicked away using capillary action. These samples were left to dry in air overnight. Imaging was undertaken on a BrukerMulitmode 8 atomic force microscope in Scanasyst (PeakForce Tapping®) mode, which is based upon tapping mode AFM, but whereby the imaging parameters are constantly optimised through the force curves that are collected, preventing damage of soft samples. Bruker Scanasyst-Air probes were used, with a spring constant of 0.4 - 0.8 N/m and a tip radius of 2 nm.

NMR measurements

Samples of **1** and **2** were prepared by dissolving the 10 mg of the appropriate peptide with 1.5 equivalents 0.1M NaOD and making the solution up to 1 mL using D_2O, giving a final pD of approximately 10. ^{1}H spectra were recorded using a Bruker Avance III HD 400 MHz NMR spectrometer.

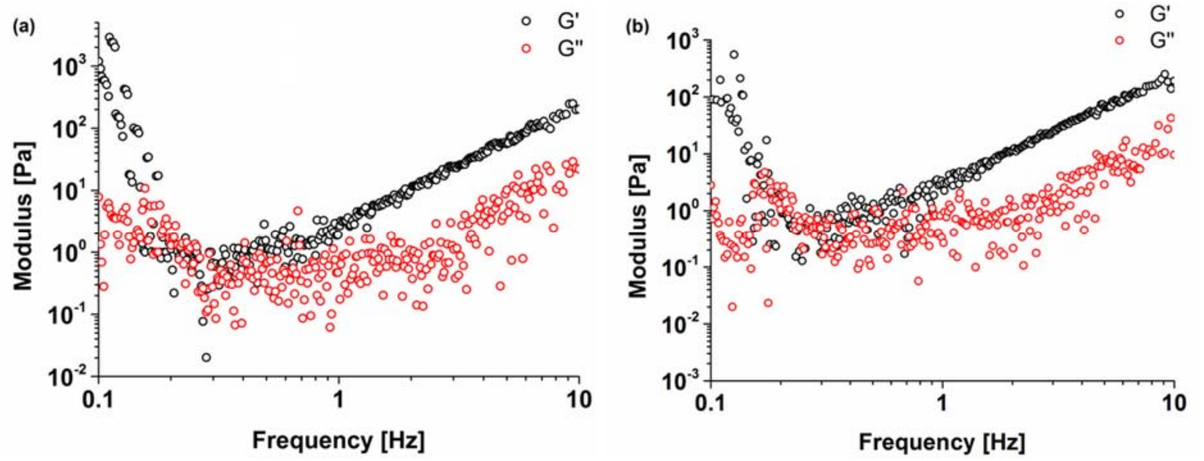

Figure S1 – Oscillatory rheology frequency sweeps of peptide sols (a) indole-diphenylalanine **1** and (b) carbazole-dihenylalanine **2** displaying typical liquid-like behaviour.

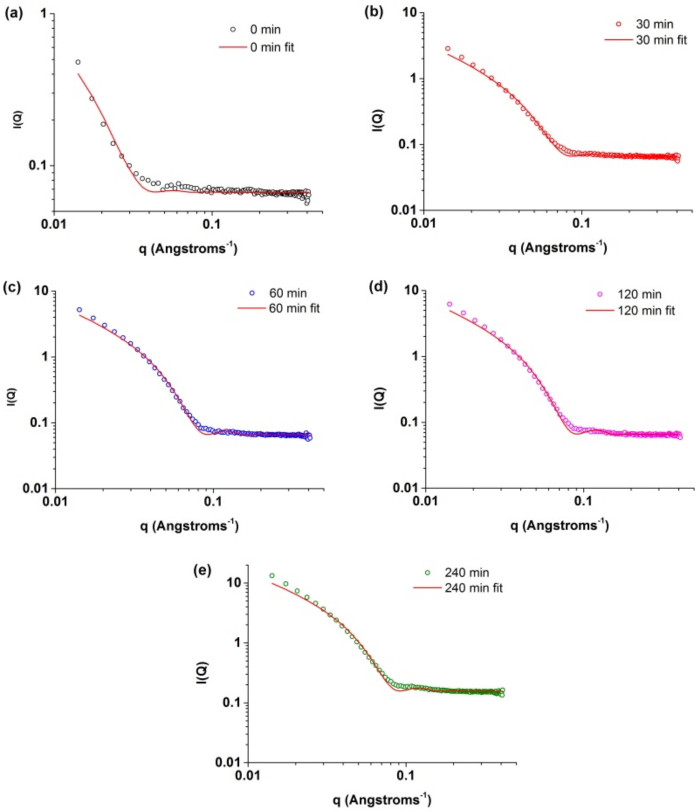

Figure S2 – Fits of SANS data to the cylindrical model selected in SASView for hydrogels of **1** at (a) 0 mins, (b) 30 mins, (c) 60 mins, (d) 120 mins and (e) 240 mins.

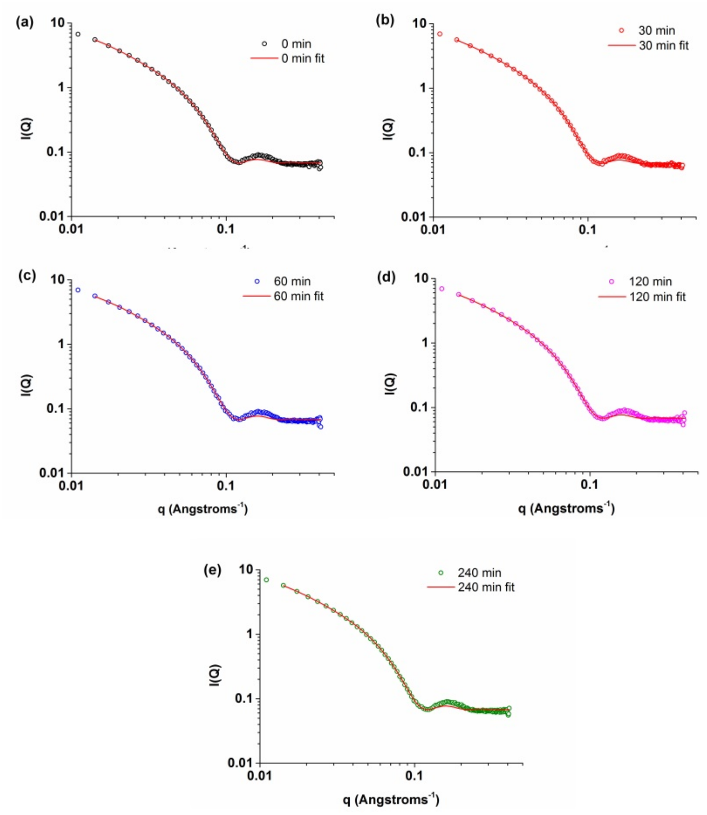

Figure S3 – Fits of SANS data to the cylindrical model selected in SASView for hydrogels of **2** at (a) 0 mins, (b) 30 mins, (c) 60 mins, (d) 120 mins and (e) 240 mins.

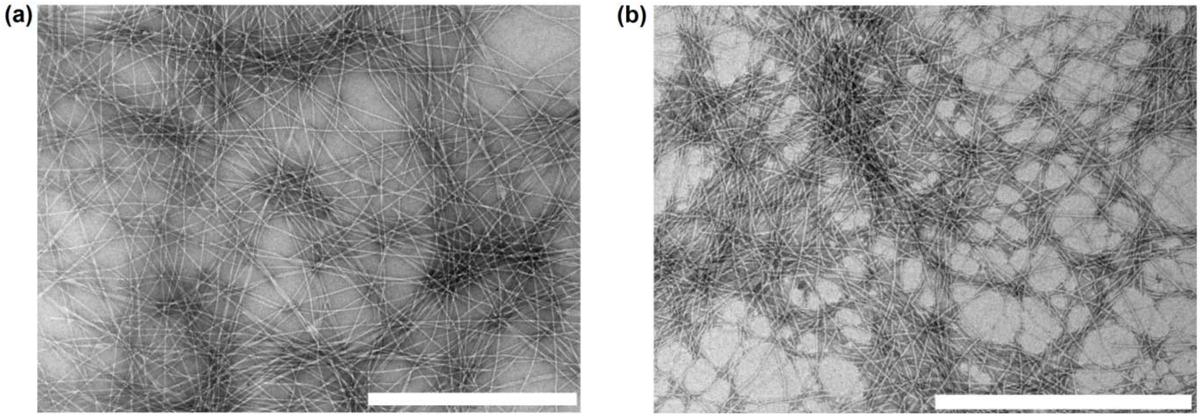

Figure S4 – Negative stain TEM images of hydrogel networks of carbazole-diphenylalanine **2** after (a) 1 h and (b) 4 h. Scale bar denotes 1 µm.

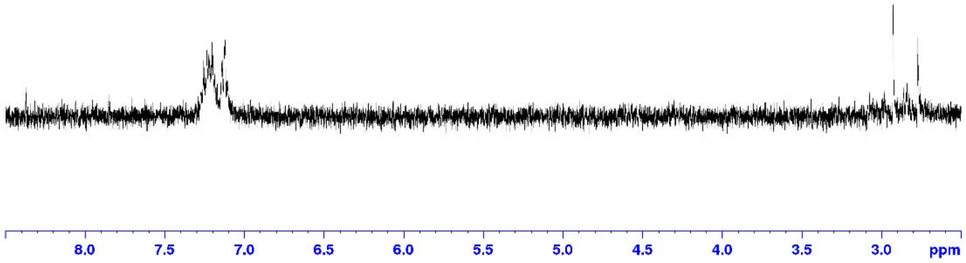

Figure S5 – ^{1}H NMR of **2** in D_2O at a pD of 9, at 1% (w/v). The spectrum is almost identical to that presented in Figure 4b, however the D_2O solvent resonance peak has been removed through excitation sculpting. The broadened signals show that there is also self-assembly present for **2** at a pD of 9.

References

1. Martin, A. D., Robinson, A. B., Mason A. F. & Thordarson, P. Exceptionally strong hydrogels through self-assembly of an indole-capped dipeptide. *Chem. Commun.* **50**, 15541-15544 (2014).
2. Martin, A. D., Robinson A. B. & Thordarson, P. Biocompatible small peptide super-hydrogelators bearing carbazole functionalities *J. Mater. Chem. B.* **3**, 2277-2280 (2015).
3. Kline, S. R. Reduction and analysis of SANS and USANS data using IGOR Pro *J. Appl. Crystallogr.* **39**, 895-900 (2006).
4. Pedersen, J. S. & Schurtenburger, P. Scattering Functions of Semiflexible Polymers with and without Excluded Volume Effects. *Macromolecules* **29**, 7602-7612 (1996).
5. *SasView for Small Angle Scattering Analysis*, http://www.sasview.org/
